# Presentation of Adult-onset Asthma and Periocular Xanthogranuloma with Intermediate Uveitis and Hodgkin's Lymphoma: A Case Report

**DOI:** 10.18502/jovr.v19i2.9629

**Published:** 2024-06-21

**Authors:** Sahba Fekri, Mohammad-Hasan Rikhtehgar, Abbas Bagheri, Amirreza Veisi, Amir A. Azari

**Affiliations:** ^1^Ophthalmic Research Center, Research Institute for Ophthalmology and Vision Science, Shahid Beheshti University of Medical Sciences, Tehran, Iran; ^2^Ocular Tissue Engineering Research Center, Research Institute for Ophthalmology and Vision Science, Shahid Beheshti University of Medical Sciences, Tehran, Iran; ^4^https://orcid.org/0000-0001-8304-3804; ^5^Sahba Fekri: https://orcid.org/0000-0002-7388-6725

**Keywords:** Adult-onset Asthma, Hodgkin Lymphoma, Intermediate Uveitis, Xanthogranuloma

## Abstract

**Purpose:**

To report a case of adult-onset asthma and periocular xanthogranuloma (AAPOX) in a patient with intermediate uveitis and a history of Hodgkin's lymphoma (HL).

**Case Report:**

A 51-year-old man with a past medical history of HL presented with blurred vision, asthma, and bilateral yellowish eyelid lesions. The eyelid lesions and asthma appeared 10 years after being diagnosed with HL. Physical examination was significant for multiple subcutaneous and firm eyelid masses in addition to the presence of pre-auricular and submandibular lymphadenopathies. Ophthalmic examination revealed bilateral intermediate uveitis and mild macular edema. Further systemic evaluations, including laboratory testing and imaging, were normal. Excisional biopsy of the eyelid lesions was performed and the histopathologic examination was consistent with the diagnosis of AAPOX.

**Conclusion:**

The presence of AAPOX in a patient with intermediate uveitis and history of HL suggests that immunological dysfunction may play a role in the pathogenesis of adult orbital xanthogranulomatous disease.

##  INTRODUCTION

Systemic or localized proliferation of non-Langerhans histiocytes with a tendency to affect of systemic involvement, they are classified into four subgroups: necrobiotic xanthogranuloma (NXG), which is the most common form; adult-onset asthma and periocular xanthogranuloma (AAPOX); Erdheim–Chester disease (ECD); and adult-onset xanthogranuloma (AOX), which is the least common form.^[1,2]^ AAPOX is a rare subtype of AOXGD and the knowledge about this condition is limited.

Herein, we report a case of AAPOX in a patient with bilateral intermediate uveitis and history of Hodgkin's lymphoma (HL) which to the best of our knowledge is presented for the first time. This association supports the role of immunologic derangement in the pathogenesis of AAPOX.

**Figure 1:**
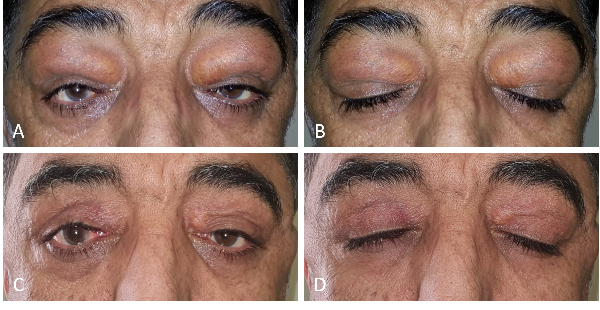
Patient's photograph shows bilateral yellowish subcutaneous lesions in the upper eyelids causing ptosis (A & B). There is neither sign of recurrence nor lagophthalmos, and the mechanical ptosis has improved significantly a year after the surgery (C & D).

**Figure 2:**
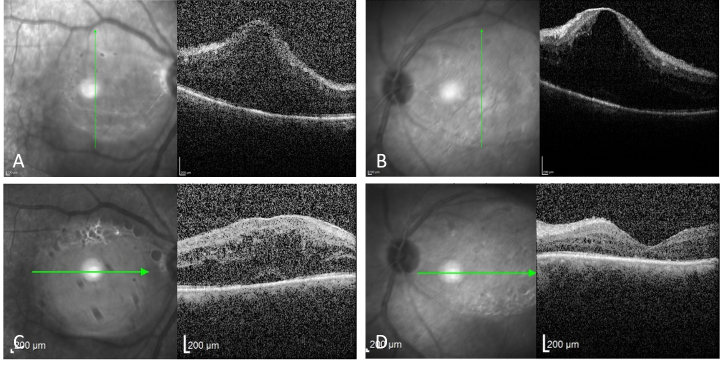
Optical coherence tomography of both eyes demonstrates severe cystoid macular edema before periocular steroid injection (A & B). A significant reduction in cystoid macular edema was observed after the injection (C & D).

**Figure 3:**
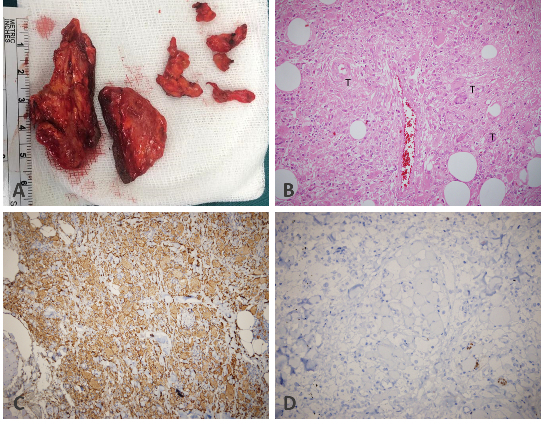
Pathological gross examination of the specimen shows yellowish-brown, firm, and non-encapsulated lesions of varying size (A). H & E ( × 40) examination of the excised lesions demonstrates foamy histiocytes and Touton giant cells along with the presence of lymphocytic infiltration and scattered eosinophils (B). Immunohistochemistry reveals histiocytes that are CD-68 positive (C), but S100 negative (D).

##  CASE REPORT

This study was approved by the scientific and ethics committees of Labbafinejad Medical Center. A written consent form was obtained from the patient publish the findings and images. A 51-year-old man presented to us with blurry vision in both eyes, bilateral periocular lesions, and asthma. He had a history of HL which had been treated with aggressive chemotherapy more than a decade before presentation without any recurrences. Approximately three years after the completion of chemotherapy, the patient had noticed small yellowish eyelid nodules along with experiencing asthma. The eyelid lesions grew very slowly during the years and mainly involved the upper eyelids. He had undergone several incisional biopsies of the eyelid lesions along with multiple imaging studies to search for a possible extranodal recurrence of HL; however, the results were inconclusive. During the past few months before presentation, he also noticed a progressive bilateral loss of vision in addition to experiencing asthma and the presence of the eyelid lesions. He had no significant systemic disorders except for the history of HL. His past surgical history was limited to the eyelid biopsies, and his family history was unremarkable. He used intermittently a combination of a salmeterol/fluticasone propionate inhaler to alleviate his respiratory symptoms. Physical examination revealed bilateral and symmetric periocular masses in the upper eyelids causing significant ptosis. These lesions were yellow in color and were firm, immobile, and non-tender when palpated [Figure 1A & 1B]. Besides the bilateral pre-auricular and submandibular enlarged lymph nodes being palpated, no other significant findings were noted on physical examination. Ophthalmic examination revealed best-corrected visual acuity of 20/80 and 20/160 for the right and left eyes, respectively, without relative afferent pupillary defect. Extraocular motilities were full and no proptosis was noted. Slit-lamp examination revealed a significant presence of 1+ cells in the anterior vitreous in both eyes. Intraocular pressure was within the normal range bilaterally. A dilated fundus examination revealed bilateral mild vitritis with significant cystoid macular edema which was further confirmed and documented by optical coherence tomography [Figure 2A & 2B]. A complete systemic workup was found to be in the normal range. Orbital CT-scan demonstrated bilateral, extraconal, homogenous, poorly circumscribed periorbital lesions with irregular borders; no evidence of bony erosions was present. The thoracic CT-scan and abdominal ultrasound were normal. A vitreous biopsy showed a non-specific chronic inflammatory reaction. The patient underwent bilateral debulking of the upper eyelid lesions. An anterior orbitotomy via the superior eyelid crease approach was performed, and multiple pieces of yellowish-brown, lobulated, non-encapsulated masses were removed with the preservation of the lacrimal glands. At the end of the surgery, 40 mg local triamcinolone acetonide was injected into the surgery site to prevent a recurrence, and a similar dosage was injected into the subtenon space to treat uveitic macular edema (UME).

Histopathological examination of the excised lesions demonstrated the presence of foamy histiocytes and Touton giant cells along with the presence of lymphocytic infiltration and scattered eosinophils, confirming the diagnosis of AAPOX. Immunohistochemical studies demonstrated the presence of many CD-68 positive histiocytes while staining for S100 and IgG4 antigens were negative [Figure 3].

One month following the surgery and injections, visual acuity and ptosis mildly improved, and the severity of vitritis and UME were significantly decreased [Figure 2B & 2C]. There was also no evidence of surgically induced lagophthalmos in our patient. The one-year follow-up examination revealed no recurrence of the periorbital lesions [Figure 1C & 1D].

##  DISCUSSION

The presence of foamy histiocytes and Touton giant cells, along with varying degrees of lymphocytic infiltration, fibrosis, and necrosis are the histopathologic hallmarks of AOXGD.^[3]^ AOXGD is classified into four subtypes based on the clinical presentations, site of involvement, and the systemic associations.^[1,3]^ Although all of these entities share similar histopathologic findings, each subtype has its own distinctive features. AOX usually presents with an isolated xanthogranulomatous lesion without any systemic involvement. Subcutaneous yellowish lesions in the eyelids and anterior orbit which tend to ulcerate and become fibrotic are characteristic for NXG. In ECD, diffuse lymphohistiocytic infiltration of the internal organs (e.g., heart and lungs), bones, central nervous system, retroperitoneal spaces, along with the involvement of the orbits are typically seen.^[1,3]^

Our patient had typical clinical and histopathological features of AAPOX. This condition affects men two times more frequently than women,^[4]^ and it presents with adult-onset asthma and bilateral yellowish periocular and eyelid lesions which slowly grow over many months to years; these lesions may extend into deeper parts of the orbit with the involvement of orbital fat, extraocular muscles, lacrimal glands, and conjunctiva.^[1,5,6]^ AAPOX may be associated with reactive lymphadenopathy and increased levels of polyclonal IgG.^[7,8]^ London et al^[7]^ have claimed that AAPOX may be a variant of an IgG4-related disease. Other reported associations are paraproteinemia, chronic lymphocytic leukemia, multiple myeloma, Burkitt's lymphoma, non-Hodgkin lymphoma, and lymphoplasmacytic sclerosing pancreatitis.^[1,3][9]^ In a review article, immune dysfunction was noted in all AAPOX cases.^[3]^ Characteristic histopathological features of AAPOX are lymphoid aggregates with germinal centers in addition to foamy histiocytes and Touton giant cells. On immunohistochemistry, histiocytes are usually positive for CD68, CD163, and factor XIIIa, but are negative for CD21, CD35, S100, and CD1a.^[1,3]^ Lymphoid cells within the germinal centers are positive for CD20 and negative for Bcl-2, and parafollicular cells are CD3 positive and are often predominantly CD8 positive.^[1,3]^ These histopathologic findings and reported associations strongly support the hypothesis that immunological dysfunction, especially in the B cell population, plays an important role in the pathogenesis of AAPOX.

The presented case had bilateral intermediate uveitis, bilateral pre-auricular, and submandibular lymphadenopathy, and HL in addition to typical clinical (bilateral yellowish periocular lesions and asthma) and histopathological features of AAPOX. To the best of our knowledge, this combination has not been previously reported in the literature. This association supports the aforementioned hypothesis that immunological dysfunction plays an important role in the pathogenesis of AAPOX.

In summary, our findings showed that AAPOX may be a manifestation of an immune system disorder. It may have an association with HL and intermediate uveitis.

##  Financial Support and Sponsorship

None.

##  Conflicts of Interest

None.

##  Declaration of Patient Consent

The authors certify that they have obtained all appropriate patient consent forms. In the form the patient has given his consent for his images and other clinical information to be reported in the journal. The patient understand that his name and initial will not be published and due efforts will be made to conceal his identity, but anonymity cannot be guaranteed.
